# The impact of segmentectomy versus lobectomy on pulmonary function in patients with non-small-cell lung cancer: a meta-analysis

**DOI:** 10.1186/s13019-022-01853-3

**Published:** 2022-05-07

**Authors:** Yuan Xu, Yingzhi Qin, Dongjie Ma, Hongsheng Liu

**Affiliations:** Department of Thoracic Surgery, Peking Union Medical College Hospital, Chinese Academy of Medical Science and Peking Union Medical College, Beijing, China

**Keywords:** Non-small-cell lung cancer, Segmentectomy, Lobectomy, Pulmonary function

## Abstract

**Objective:**

Segmentectomy has been reported as an alternative to lobectomy for small-sized NSCLC without detriment to survival. The long-term benefits of segmentectomy over lobectomy on pulmonary function have not been firmly established. This meta-analysis aims to compare postoperative changes in pulmonary function in NSCLC patients undergoing segmentectomy or lobectomy.

**Methods:**

Medline, Embase, Web of Science and Scopus were searched through March 2021. Statistical comparisons were made when appropriate.

**Results:**

Fourteen studies (2412 participants) out of 324 citations were included in this study. All selected studies were high quality, as indicated by the Newcastle–Ottawa scale for assessing the risk of bias. Clinical outcomes were compared between segmentectomy and lobectomy. ΔFEV1 [10 studies, *P* < 0.01, WMD = 0.40 (0.29, 0.51)], ΔFVC [4 studies, *P* < 0.01, WMD = 0.16 (0.07, 0.24)], ΔFVC% [4 studies, *P* < 0.01, WMD = 4.05 (2.32, 5.79)], ΔFEV1/FVC [2 studies, *P* < 0.01, WMD = 1.99 (0.90, 3.08)], and ΔDLCO [3 studies, *P* < 0.01, WMD = 1.30 (0.69, 1.90)] were significantly lower in the segmentectomy group than in the lobectomy group. Subgroup analysis showed that in stage IA patients, the ΔFEV1% [3 studies, *P* < 0.01, WMD = 0.26 (0.07, 0.46)] was significantly lower in the segmentectomy group. The ΔDLCO% and ΔMVV% were incomparable.

**Conclusion:**

Segmentectomy preserves more lung function than lobectomy. There were significantly smaller decreases in FEV1, FVC, FVC%, FEV1/FVC and DLCO in the segmentectomy group than in the lobectomy group.

**Supplementary Information:**

The online version contains supplementary material available at 10.1186/s13019-022-01853-3.

## Introduction

Lung cancer is one of the leading cause of cancer-related death worldwide [1]. Surgical resection for non-small-cell lung cancer (NSCLC) is the standard treatment that leads to the best chance of a cure. For the 100-year history of surgery, lobectomy has remained the gold standard for operable NSCLC. In recent years, segmentectomy has been reported as an alternative to lobectomy for small-sized NSCLC without detriment in survival [2]. Theoretically, segmentectomy has an advantage over lobectomy on anatomical functional. However, the long-term benefits of segmentectomy over lobectomy on pulmonary function have not been firmly established. Reports related to the utility of segmentectomy in preserving lung function are conflicting. The purpose of this study was to perform a meta-analysis to compare postoperative changes in pulmonary function in NSCLC patients undergoing segmentectomy or lobectomy.

## Methods

### Inclusion and exclusion criteria

Studies were included if they met the following inclusion criteria: (1) patients diagnosed with NSCLC underwent surgical treatment; (2) comparative data between segmentectomy and lobectomy were available; and (3) preoperative and postoperative pulmonary function data were available. The outcomes included forced expiratory volume in 1 s (FEV1), predicted FEV1 percentage (FEV1%), forced vital capacity (FVC), predicted FVC percentage (FVC%), FEV1/FVC, maximal voluntary ventilation (MVV), diffusion capacity of carbon monoxide (DLCO) and predicted DLCO percentage (DLCO%). Studies were excluded if the full text was not in English or could not be accessed.

### Search strategy

Medline, Embase, Web of Science and Scopus were searched through May 2021. The following search terms and strategies were used: (1) respiratory function OR pulmonary function OR FEV1 OR FVC OR MVV OR DLCO; (2) lung cancer; (3) lobectomy AND (segmentectomy OR sublobar resection OR limited resection), and (1) AND (2) AND (3). Data were extracted with a standardized form. The Newcastle–Ottawa Scale (NOS) was used for quality assessment.

### Statistical analysis

Inconsistency between studies was quantified by calculating the I^2^ statistic. Continuous variables were reported as weighted mean differences (WMDs) and 95% confidence intervals (95% CIs). A random-effects model was used for heterogeneous data (I^2^ > 50%), whereas a fixed-effects model was used for homogenous data (I^2^ < 50%). *P* < 0.05 was considered to be statistically significant. SAS software, version 9.1 (SAS Institute, Cary, NC, USA) and Review Manager, version 5.4 (The Cochrane Collaboration) were used to perform statistical analysis.

## Results

### Search results

**Fig. 1:**
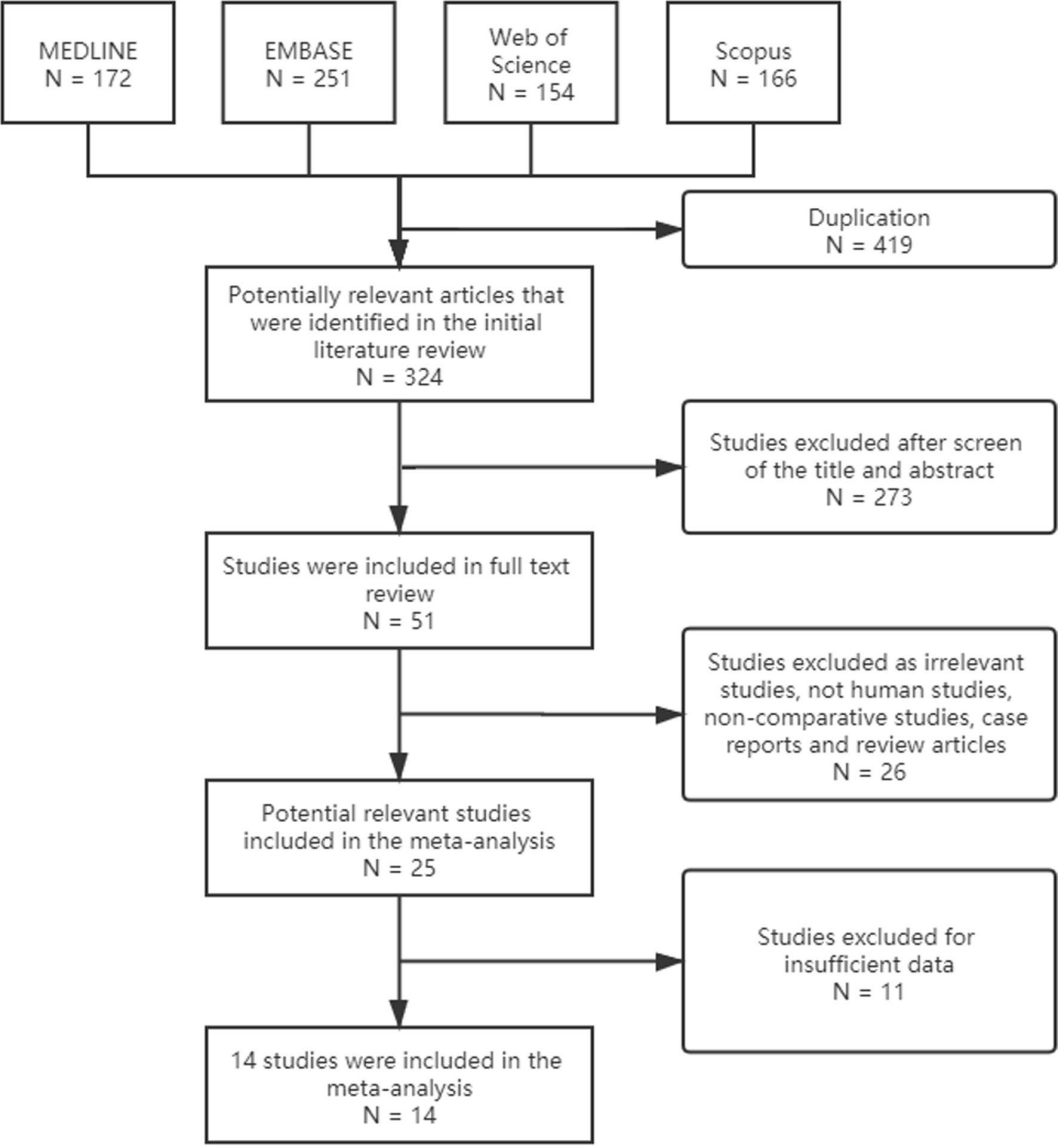
A flow diagram of the study selection

The initial database search identified 172 articles in Medline, 251 in Embase, 154 in Web of Science and 166 in Scopus. After excluding duplicate records, 324 studies were included. A total of 273 articles were excluded because they failed to meet the inclusion criteria after review of the abstracts and titles. An additional 37 articles were excluded after the full text review. Hence, a total of 14 studies (13 retrospective and 1 prospective observational) including 2412 patients (976 sublobar resection and 1436 lobectomy) were finally selected. The detailed selection process is shown in Fig. 1.

The major characteristics of the participants in the included studies are shown in Table 1. Patients in 8 studies had NSCLC with TNM stage I. Six studies included the VATS approach only, 2 included the open approach only, and 3 included both. Two studies included wedge resections. The follow-up time (from surgery to postoperative pulmonary function) ranged from 3 to 60 months.

**Table 1:** Basic characteristics of the included studies

ID	Location	Enrolment Year	Total No	No. of segmentectomy	TNM stage	Approach	Wedge resection included	Involved lobe			Follow-up (month)
								Upper	Middle	Lower	
Kim 2015 [3]	Korea	2003–2012	300	73	I–IV	VATS	yes	NM			3 and 12
Zhong 2020 [4]	China	2014–2016	144	68	IA–IB	VATS	no	35	9	24	6
								37	11	28	
Nomori 2018 [5]	Japan	2013–2016	206	103	NM	VATS + open	no	57	0	46	6
								57	0	46	
Takizawa 1999 [6]	Japan	1993–1996	80	40	IA	open	no	25	0	15	12
								28	0	12	
Tane 2019 [7]	Japan	2012–2017	148	74	IA1–IA2	VATS	no	45	0	29	6
								45	0	29	
Helminen 2020 [1]	Japan	2007–2019	215	105	I–III	VATS	no	NM			NM
Gu 2018 [8]	China	2011–2014	109	34	IA	VATS	yes	23	0	11	6
								50	0	25	
Kobayashi 2017 [9]	Japan	2001–2009	346	118	I–III	VATS + open	no	NM			12 & 60
Keenan 2004 [10]	USA	1996–2001	201	54	IA	NM	no	23	0	31	12
								87	17	43	
Kashiwabara 2009 [11]	Japan	2000–2006	118	71	I	NM	no	38	0	33	6
								32	0	15	
Macke 2015 [12]	USA	1996–2001	159	77	I	VATS + open	no	36	9	32	6–36
								48	0	34	
Saito 2014 [13]	Japan	2006–2012	178	52	I	open	no	30	0	22	6
								71	6	49	
Yoshimoto 2010 [14]	Japan	2005–2008	20	13	NM	NM	no	13	0	0	5
								7	0	0	
Hwang 2015 [15]	Korea	2005–2013	188	94	I–IV	VATS	no	NM			NM

*NM* not mentioned, *VATS* video-assisted thoracoscopic surgery

### Quality assessment

The quality of the included studies was assessed using the NOS (www.ohri.ca/programs/clinical_epidemiology/oxford.htm). Two independent reviewers conducted the assessment. Disagreements were resolved by discussion. Of the studies, seven scored 9 points, four scored 8 points, two scored 7 points, and one scored 6 points, indicating that all the studies had relatively high quality (Table 2).

**Table 2:** Quality assessment according to the Newcastle–Ottawa scale

ID	Selection	Comparability	Exposure	Total score
Kim 2015 [3]	3	2	3	8
Zhong 2020 [4]	4	2	2	8
Nomori 2018 [5]	4	2	3	9
Takizawa 1999 [6]	4	2	3	9
Tane 2019 [7]	4	2	3	9
Helminen 2020 [1]	3	2	2	7
Gu 2018 [8]	4	2	3	9
Kobayashi 2017 [9]	4	2	3	9
Keenan 2004 [10]	2	1	3	6
Kashiwabara 2009 [11]	4	2	3	9
Macke 2015 [12]	4	2	3	9
Saito 2014 [13]	4	1	3	8
Yoshimoto 2010 [14]	3	2	2	7
Hwang 2015 [15]	4	2	2	8

### Clinical outcomes

#### FEV1 and FEV1%

**Fig. 2:**
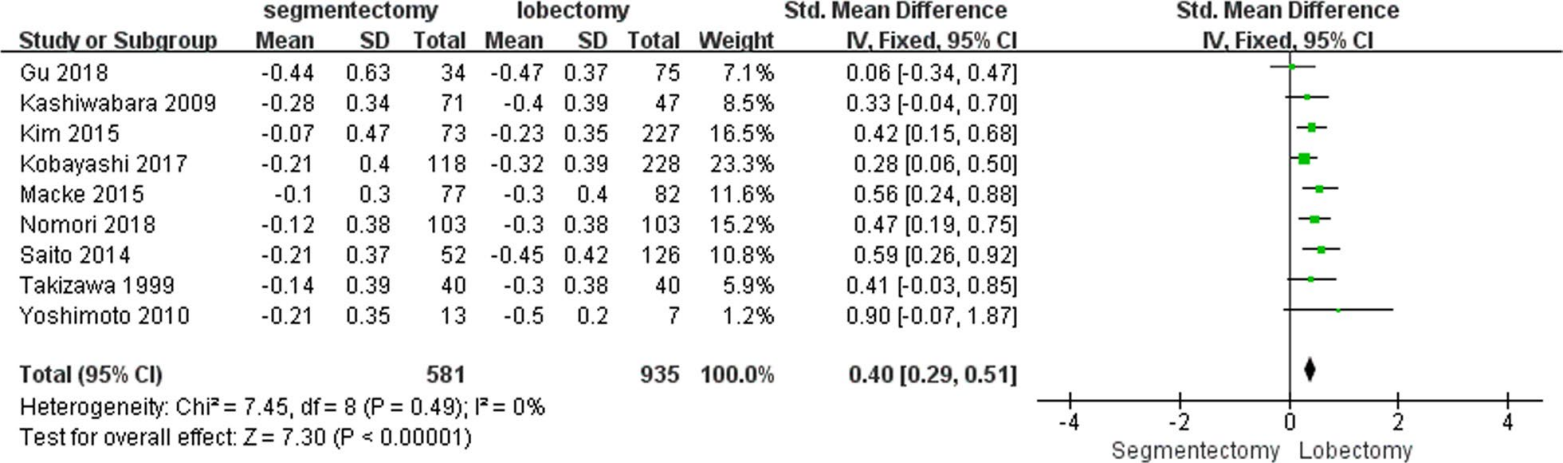
Weighted ΔFEV1 between the segmentectomy group and the lobectomy group

FEV1 was the most frequently reported functional value. It was recorded in 10 studies (n = 1664, I^2^ = 95%, random-effects model, Additional file 1: Fig. S1). The mean ΔFEV1 varied from − 0.10 to − 0.44 (segmentectomy group) and − 0.23 to − 0.50 (lobectomy group). After ruling out one study with high heterogeneity [7], the ΔFEV1 was significantly lower in the segmentectomy group than in the lobectomy group [*P* < 0.01, WMD = 0.40 (0.29, 0.51); heterogeneity: Chi^2^ = 7.45, df = 8, *P* = 0.49; I^2^ = 0%, fixed-effects model; Fig. 2].

**Fig. 3:**
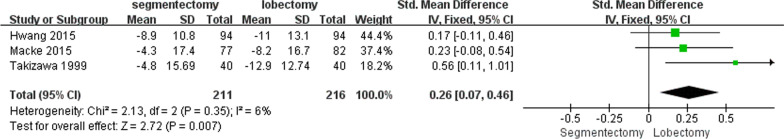
Weighted ΔFEV1% between the segmentectomy group and the lobectomy group in Stage IA patients

The FEV1% was incomparable due to the high heterogeneity (8 studies, n = 1633, I^2^ = 96%, random-effects model, Additional file 2: Fig. S2). The mean ΔFEV1% varied from − 9.2 to + 1.0 (segmentectomy group) and − 16.2% to − 8.1% (lobectomy group). Subgroup analysis showed that in stage IA patients, the ΔFEV1% was significantly lower in the segmentectomy group [3 studies, n = 427; *P* < 0.01, WMD = 0.26 (0.07, 0.46); heterogeneity: Chi^2^ = 2.13, df = 2, *P* = 0.35; I^2^ = 6%, fixed-effects model; Fig. 3].

#### FVC and FVC%

**Fig. 4:**
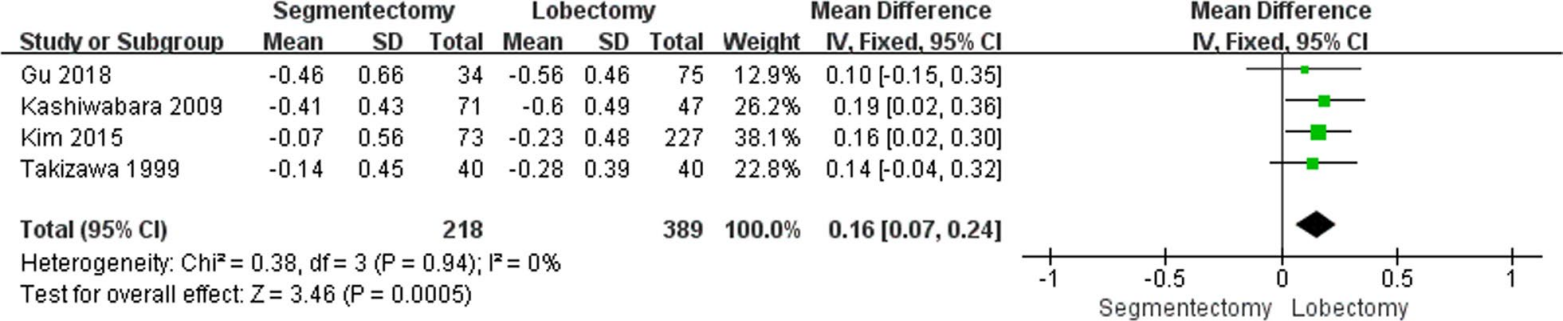
Weighted ΔFVC between the segmentectomy group and the lobectomy group

Four studies (n = 607) provided FVC values. The mean ΔFVC varied from − 0.07 to − 0.46 (segmentectomy group) and − 0.23 to − 0.6 (lobectomy group). The ΔFVC was significantly lower in the segmentectomy group than in the lobectomy group [*P* < 0.01, WMD = 0.16 (0.07, 0.24); heterogeneity: Chi^2^ = 0.38, df = 3, *P* = 0.94; I^2^ = 0%, fixed-effects model; Fig. 4].

**Fig. 5:**
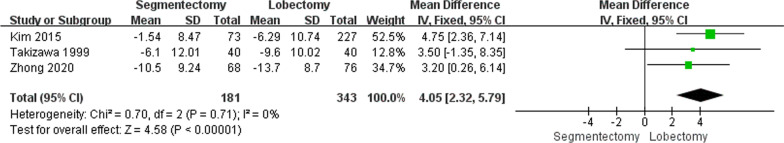
Weighted ΔFVC% between the segmentectomy group and the lobectomy group

The FVC% was reported in 4 studies (n = 725, I^2^ = 79%, random-effects model, Additional file 3: Fig. S3). After ruling out one study with high heterogeneity [10], the ΔFVC% was significantly lower in the segmentectomy group than in the lobectomy group [*P* < 0.01, WMD = 4.05 (2.32, 5.79); heterogeneity: Chi^2^ = 0.70, df = 2, *P* = 0.71; I^2^ = 0%, fixed-effects model; Fig. 5]. The ΔFVC% varied from − 1.5 to − 10.5% in the segmentectomy group and − 4.4% to − 13.7% in the lobectomy group.

### Other outcomes

**Fig. 6:**
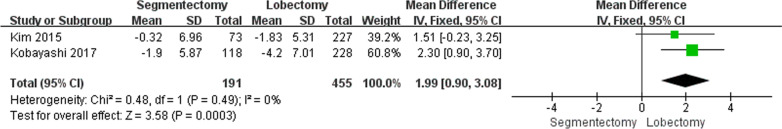
Weighted ΔFEV1/FVC between the segmentectomy group and the lobectomy group

The ΔFEV1/FVC was significantly lower in the segmentectomy group than in the lobectomy group [2 studies, n = 646; *P* < 0.01, WMD = 1.99 (0.90, 3.08); heterogeneity: Chi^2^ = 0.48, df = 1, *P* = 0.49; I^2^ = 0%, fixed-effects model; Fig. 6]. The ΔFEV1/FVC varied from − 0.3 to − 1.9 in the segmentectomy group and − 1.8 to − 4.2 in the lobectomy group.

**Fig. 7:**
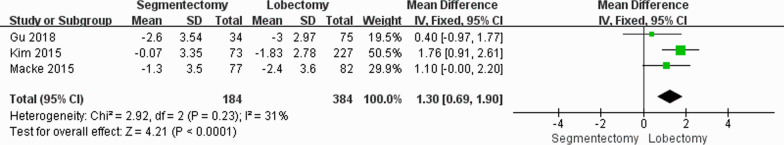
Weighted ΔDLCO between the segmentectomy group and the lobectomy group

Similarly, the ΔDLCO was significantly lower in the segmentectomy group [3 studies, *P* < 0.01, WMD = 1.30 (0.69, 1.90); heterogeneity: Chi^2^ = 2.92, df = 2, *P* = 0.23; I^2^ = 31%, fixed-effects model; Fig. 7]. The ΔDLCO varied from − 0.07 to − 2.6 in the segmentectomy group and − 1.8 to − 3 in the lobectomy group.

The ΔDLCO% (n = 660, I^2^ = 96%, random-effects model, Additional file 4: Fig. S4) and ΔMVV% (n = 345, I^2^ = 96%, random-effects model, Additional file 5: Fig. S5) were incomparable.

## Discussion

Previous studies suggested that segmentectomy confers little functional advantage over lobectomy [10]. It was concluded that lobectomy should remain the procedure of choice despite the slight functional advantage of limited resection. In the present study, we compared postoperative changes in pulmonary function in patients undergoing segmentectomy or lobectomy. This meta-analysis showed that there were significantly fewer decreases in FEV1, FVC, FVC%, FEV1/FVC and DLCO in the segmentectomy group than in the lobectomy group. Subgroup analysis also showed that the decrease in FEV1% was significantly less in the segmentectomy group in stage IA patients. Altogether, these studies support the assumption that segmentectomy preserves more lung function than lobectomy.

Pulmonary function tests are recommended in all patients who undergo thoracic surgery [16]. Theoretically, segmentectomy has an anatomical functional advantage over lobectomy. First, as the adult lung cannot regenerate new alveolar septal tissues, postoperative pulmonary function is mainly determined by the amount of lung resected. Second, anatomical excursion of the nonoperated lobe after lobectomy occurred. For example, a right upper lobectomy will damage the function of the middle lobe due to the kink of the middle lobar bronchus and pulmonary artery [17]. Third, compensatory lung growth could already have occurred in the ipsilateral nonoperated lobe in the lobectomy group before the operation due to the decreased function in the operated lobe, resulting in less space for postoperative lung growth [5].

FEV1 is an indicator of airway resistance. Changes in FEV1 are largely related to ventilation mechanisms, including existing airway obstruction, compensatory expansion of the residual lung, and chest wall activity [18]. Lung resection will inevitably lead to displacement of the remaining lobe. The meta-analysis showed that the decrease in FEV1 was higher in the lobectomy group, indicating that lobectomy is more likely to increase airway resistance. A recent meta-analysis conducted in early-stage NSCLC patients concluded that segmentectomy conserves more FEV1 (5 studies, 933 patients), %FEV1 (5 studies, 976 patients) than lobectomy (28). Changes in the FVC are mainly determined by the amount of lung tissue resected. After lung resection, the remaining part of the lung expands and compensates for the resected lobe [19]. The meta-analysis showed that both FVC and FVC% were more rapidly improved in the segmentectomy group, indicating that segmentectomy has an advantage in the preservation of lung volume. FEV1/FVC is an essential parameter to phenotype the functional pattern of patients if obstructive, restrictive or normal [20]. There were only 2 studies reporting changes in FEV1/FVC. The meta-analysis showed that the ΔFEV1/FVC was lower in the segmentectomy group. DLCO reflects the capillary surface area available for gas diffusion. Preoperative DLCO has been demonstrated to predict the risk of complications, short- and long-term outcomes and the length of hospitalization in patients undergoing thoracic surgery [21]. The meta-analysis showed a lower degree of DLCO decrease in the segmentectomy group, indicating that it had better preservation of oxygenation.

Pulmonary function after lung resection can be affected by a number of factors. The number of resected segments is an important factor. Several studies observed a positive relationship between the number of resected segments and the loss of pulmonary function [5, 12, 22]. As each lobe consisted of different numbers of segments, the improvement of pulmonary function was also determined by the resected lobe. Therefore, Macke et al. classified patients into the following two groups: those who had 1–2 segments resected and those who had 3–5 segments resected [12]. This classification could reduce the influence of different lobes and could be adopted in future studies. Furthermore, anatomical excursion of the remaining lobe could also influence the preservation rate of the residual lobe. As mentioned above, right upper lobectomy can cause a reduction in the volume of the right middle lobe [23]. Tane et al. found that residual lobe function was the most preserved after S6 segmentectomy, suggesting that the shape of the preserved segments (basal segment) may be amenable to inflation without anatomic displacement [7].

Emphysema could also affect postoperative pulmonary function. Kashiwabara et al. reported that there were some patients with emphysema receiving lobectomy who had a greater advantage in postoperative pulmonary functions than segmentectomy [11]. It was speculated that the removal of an emphysematous parenchyma may have caused a partial improvement of the regional lung volume distribution and ventilation inhomogeneity, thus causing 'compensatory lung growth'. However, the selected studies rarely described whether they included patients with emphysema or chronic obstructive pulmonary disease (COPD), which might result in increased heterogeneity.

The influence of the surgical approach on pulmonary function is controversial. Some researchers reported that no differences were found between VATS surgery and open surgery [24, 25]. In contrast, some studies showed low functional loss after VATS segmentectomy, indicating that the functional benefit of segmentectomy may add to that of VATS [4, 6, 15]. The selected studies in this meta-analysis contained both VATS and open approaches. Subgroup analysis failed due to the high heterogeneity. More data are needed to achieve a convincing conclusion.

The influence of follow-up time on the recovery of pulmonary function was small. Koike et al. showed that postoperative VC and FEV1 gradually increased within 3 months of surgery and remained stable thereafter [26]. Similarly, Kobayashi et al. found that the VC% and FEV1% remained almost the same 1 year after surgery [9]. It was suggested that the decreases in VC and FEV1 are caused by ageing and are not affected by the operation [27].

Our study has several limitations. The lack of prospective studies influences the data quality. In addition, several factors (e.g., smoking status, complications, surgical procedure, pathological type, adjuvant therapy, and patient effort in pulmonary function tests) that may influence pulmonary function were not included in the selected studies, adding to the heterogeneity. Third, only English literature was included in our study. We also found several articles written in Japanese, Turkish or Chinese when searching for studies. This meta-analysis may be more broadly representative if we include studies in all languages.

## Conclusion

This meta-analysis suggests that segmentectomy preserves more lung function than lobectomy. There were significantly smaller decreases in FEV1, FVC, FVC%, FEV1/FVC and DLCO in the segmentectomy group than in the lobectomy group. Therefore, segmentectomy can be regarded as an alternative therapy for NSCLC.

## Supplementary Information

**Additional file 1: Fig. S1.** Weighted ΔFEV1 between the segmentectomy group and the lobectomy group.

**Additional file 2: Fig. S2.** Weighted ΔFEV1% between the segmentectomy group and the lobectomy group.

**Additional file 3: Fig. S3.** Weighted ΔFVC% between the segmentectomy group and the lobectomy group.

**Additional file 4: Fig. S4.** Weighted ΔDLCO% between the segmentectomy group and the lobectomy group.

**Additional file 5: Fig. S5.** Weighted ΔMVV% between the segmentectomy group and the lobectomy group.

## Data Availability

The data are available on request.
