# Supplementary figures and images for: The impact of segmentectomy versus lobectomy on pulmonary function in patients with non-small-cell lung cancer: a meta-analysis
Source: J Cardiothorac Surg. 2022 May 7;17:107. doi: 10.1186/s13019-022-01853-3 (PMC9077940; doi:10.1186/s13019-022-01853-3)

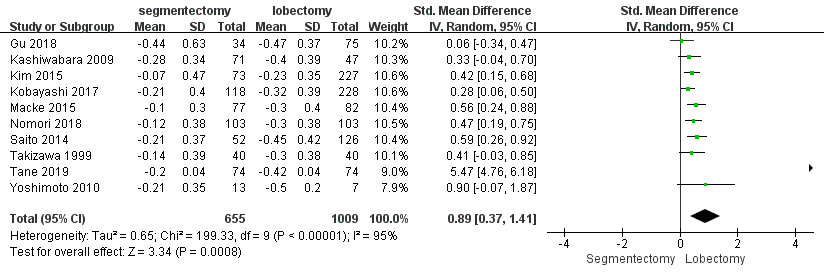

Supplement: Supplementary file 1 — Additional file 1: Fig. S1. Weighted ΔFEV1 between the segmentectomy group and the lobectomy group. [file 13019_2022_1853_MOESM1_ESM.png]

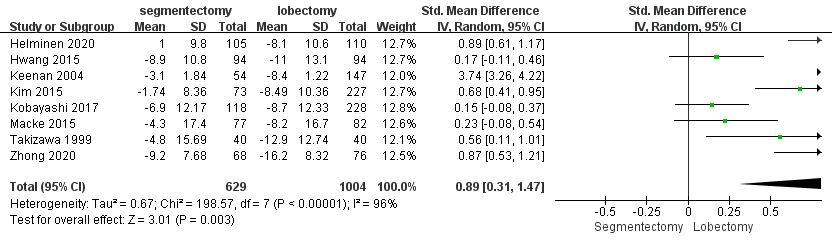

Supplement: Supplementary file 2 — Additional file 2: Fig. S2. Weighted ΔFEV1% between the segmentectomy group and the lobectomy group. [file 13019_2022_1853_MOESM2_ESM.png]

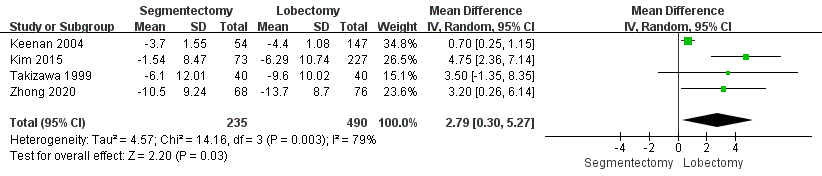

Supplement: Supplementary file 3 — Additional file 3: Fig. S3. Weighted ΔFVC% between the segmentectomy group and the lobectomy group. [file 13019_2022_1853_MOESM3_ESM.png]

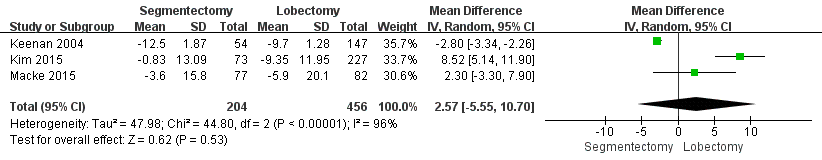

Supplement: Supplementary file 4 — Additional file 4: Fig. S4. Weighted ΔDLCO% between the segmentectomy group and the lobectomy group. [file 13019_2022_1853_MOESM4_ESM.png]

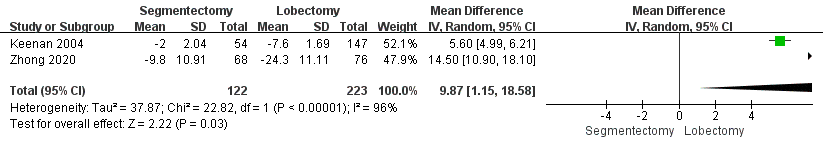

Supplement: Supplementary file 5 — Additional file 5: Fig. S5. Weighted ΔMVV% between the segmentectomy group and the lobectomy group. [file 13019_2022_1853_MOESM5_ESM.png]
